# Correlation Between Social Support, Patient Satisfaction, and Associated Factors in Patients with Schizophrenia

**DOI:** 10.7759/cureus.81222

**Published:** 2025-03-26

**Authors:** Chooni Lal, Mohammad Sabeeh Ul Haq, Farkhunda A Jaleel, Dua N Jawed

**Affiliations:** 1 Psychiatry and Behavioral Sciences, Jinnah Postgraduate Medical Centre, Karachi, PAK; 2 Medicine, Sindh Medical College, Jinnah Sindh Medical University, Karachi, PAK; 3 Pulmonology, Critical Care, Institute of Medical Technology, Jinnah Sindh Medical University, Karachi, PAK

**Keywords:** mspss, patient’s satisfaction, perceived social support, psq-18, psychiatry and mental health, schizophrenia

## Abstract

Objective

Schizophrenia is a chronic psychiatric disorder comprising cognitive, behavioral, and often emotional dysfunction. It is often accompanied by limited social support, which plays a crucial role in not only enhancing treatment adherence and resilience but also overall patient satisfaction. Given the growing recognition of perceived social support as a key factor in improving patient satisfaction, this study aims to find the correlation between perceived social support and patient satisfaction among patients diagnosed with schizophrenia who are admitted to the inpatient (wards) as well as attending the OPDs at the Department of Psychiatry, Jinnah Postgraduate Medical Centre (JPMC), Karachi, Pakistan. The study also aims to capture associated factors that may influence both of these variables.

Materials and methods

A cross-sectional study was conducted at JPMC on 151 patients with schizophrenia using convenience sampling. The data were collected using a structured questionnaire including three parts: a socio-demographic form, the Multidimensional Scale of Perceived Social Support (MSPSS), and the Patient Satisfaction Questionnaire Short Form (PSQ-18). IBM SPSS Statistics software version 20 (IBM Corp., Armonk, NY) was used for analysis. Non-parametric tests were used, including Mann-Whitney U and Kruskal-Wallis for associations and Spearman’s rho to analyze the correlation between perceived support and patient satisfaction.

Results

The sample (N = 151) comprised 62 (41.1%) females and 89 (58.9%) males. Among the study group, 53.6% (N = 81) of the patients were 18-30 years old, 64.9% (N = 98) were single, and 60.9% (N = 92) had an education level of primary or lower; 51.6% (N = 78) of patients had been diagnosed for over six years, and 71.4% (N = 108) had regular family contact. The MSPSS scores indicated moderate perceived support (39.28 ± 17.74), highest from family (3.88 ± 1.87) and lowest from friends (2.11 ± 1.68). The PSQ-18 scores were highest for interpersonal manner (3.70 ± 1.21) and lowest for general satisfaction (3.17 ± 1.09). The department of concern (ward/OPD) had significant associations with MSPSS and PSQ-18 scores, whereas gender was only found to have associations with PSQ-18 scores. Frequency of visits from family and friends was greatly associated with both MSPSS and PSQ-18 scores. Spearman’s rho showed significant positive correlations (indicated by p-value ≤ 0.05) between MSPSS (total, family, and significant others) and all PSQ-18 subsections (ρ = 0.334-0.591), while support from friends correlated only with interpersonal manner (ρ = 0.272).

Conclusion

This study highlights the impact of social support on patient satisfaction in schizophrenia. Family and significant others played a key role, while support from friends was limited but associated with interpersonal communication. Despite low or moderate perceived support among the majority of the patients, satisfaction levels remained moderate overall, suggesting other contributing factors. That being said, greater family involvement and social participation were strongly associated with both perceived support and patient satisfaction. Also, perceived support overall as well as perceived support specifically from family and significant others was found to have positive correlations with all aspects of patient satisfaction. These findings emphasize that strengthening social networks and support systems may further enhance patient experiences and care outcomes.

## Introduction

Schizophrenia is a chronic psychological illness characterized by a number of symptoms, including hallucinations, delusions, disorganized thinking, abnormal motor behavior (e.g., catatonia), and negative symptoms (lack of expression, reduced speech, etc.) [1]. It is important to approach schizophrenia in a multifaceted way by considering both biological and environmental factors [2]. For instance, systematic involvement of family members in the management of schizophrenic patients has been shown to yield positive outcomes when the family members displayed an understanding of the patient’s needs and hardships [3]. Extending beyond direct family members, social support in general, which includes not only family members but also partners and friends, has demonstrated positive correlations with the quality of life among patients suffering from schizophrenia [4]. The promotion of psychological resilience, involving a patient’s ability to adapt and adjust in the face of adverse situations, has also been associated with a good social support system [5]. It is important to note that social support goes further beyond behavioral therapy, as schizophrenic patients with increased social support have displayed better adherence to their medications as well [6, 7].

The overall importance of patient satisfaction is ultimately reflected in its correlation with patient trust, which ultimately can act as a mediator to patient loyalty and subsequent improvement of patient management [8]. While following treatment protocol is important, making sure patients have a solid perceived satisfaction with their treatment is also a relevant factor in patient management. For instance, a study conducted on patients with brain tumors reported that patients overall felt better informed regarding their treatment when they were provided with consultation recordings in advance, displaying that seemingly small adjustments in care can help to improve patient experience [9]. The association of several factors with hospital satisfaction, including satisfaction with staff, rooms, clinics, meals, etc., has been studied in the past, which displayed satisfaction with staff as the most influential factor. This emphasizes its need for attention among various staff working in any hospital [10]. Other factors such as age, gender, and ethnicity have been associated with general satisfaction, whereas factors including religion and occupation were associated with different dimensions of satisfaction such as interpersonal manner, accessibility, and convenience etc. [11].

With regards to psychiatry, patient satisfaction in psychiatric patients has been related to aspects of social support, showing an increased satisfaction among patients who received support for personal recovery [12]. Another study conducted on elderly patients receiving home health nursing reported that increased satisfaction was associated with a number of factors, one of which was social support [13]. From the aforementioned studies, although it seems that there has been some research on the association of perceived satisfaction and social support, there appears to be more room for investigation. Although when speaking generally psychiatric patients may show a positive relation between these two factors, it is also important to delve into the association between patient satisfaction and perceived social support in a condition-specific manner. Schizophrenia is distinct from some other psychiatric disorders due to its tendency to cause deficits in different cognitive domains, including social cognition, which would logically influence such a patient's ability to perceive social support [14]. This justifies investigating the relation between perceived social support and patient satisfaction among patients suffering from schizophrenia in particular. Perhaps a better understanding from this study could help shape future interventions such as psychotherapy and community-based interventions among patients.

## Materials and methods

Study design

In light of feasibility and to establish preliminary associations, a cross-sectional study was conducted in the Department of Psychiatry at Jinnah Postgraduate Medical Centre (JPMC), Karachi, Pakistan, from January 2025 to March 2025. JPMC was chosen as the study site due to its status as one of the largest and most established tertiary care hospitals in Karachi, housing a well-developed psychiatry department with extensive clinical experience and highly qualified specialists. A sample size of 145 was calculated using OpenEpi 3.01 (The OpenEpi Project, Atlanta, GA) [15] based on the total number of new patients coming to the department within the time frame of the study. A confidence interval of 95% was used, and to account for any possible missed error, the final sample was increased to 151. Approval for the study was provided by the institutional review board (IRB) at JPMC (NO.F. 2-81/2024-GENL/178/JPMC). Inclusion criteria for the sample required proper comprehension of the data collection form as well as informed consent. Patients were also required to be 18 or older and diagnosed (as per licensed psychiatrists and clinical records) with schizophrenia. Patients from both inpatient (wards) and OPD settings were recruited. Patients with an unconfirmed diagnosis/diagnosis apart from schizophrenia, or under 18, or those who did not provide consent or were unable to comprehend the data collection form were excluded from the study; incomplete forms were also excluded.

Data collection

Data collection was conducted using the non-probability convenience method. The data collection tool consisted of a structured questionnaire containing three parts: a socio-demographic form, the Multidimensional Scale of Perceived Social Support (MSPSS), a 12-item scale used as a tool to measure overall perceived social support as well as support from family, friends, and significant others separately [16], and the Patient Satisfaction Questionnaire Short Form (PSQ-18), an 18-item scale used to measure patient satisfaction across key dimensions including general satisfaction, technical quality, financial aspects, time spent with doctor communication, interpersonal manner, and accessibility and convenience [17]. To ensure validity in our study, the MSPSS and PSQ-18 scales were translated from English into Urdu and then back to English with subsequent cross-checking. Urdu versions of both scales have been validated in the past [18, 19]. The final questionnaire was administered in Urdu (the aforementioned cross-checked version) and filled out by schizophrenic patients in the wards and OPD; comprehension was ensured through proper supervision and confirmation by data collectors (via direct questioning).

Data analysis

Data analysis was conducted via IBM SPSS Statistics software, version 20 (IBM Corp., Armonk, NY). Categorical variables were described using percentages, whereas continuous variables were described using the mean with standard deviation. The test for normality (via Kolmogorov-Smirnov and Shapiro-Wilk tests) concluded that the data were not normally distributed; hence, non-parametric tests, including Mann-Whitney U and Kruskal-Wallis, were used to find associations between various socio-demographic variables and MSPSS/PSQ-18 scores. Spearman’s rho was used to assess the correlation between perceived social support and patient satisfaction.

## Results

Socio-demographic characteristics of the sample

The total sample of 151 patients consisted of 62 (41.05%) females and 89 (58.95%) males. The most common age group was 18-30 years old (N = 81, 53.6%). The proportion of patients who answered the questionnaire from the inpatient setting (i.e., ward) (N = 78, 51.6%) was similar to the proportion of patients who answered while visiting the outpatient setting (i.e., OPD) (N = 73, 48.3%). The majority of the patients (N = 98, 64.9%) were single. Most patients (60.9%) had received a maximum education of primary (i.e., up to fifth grade) or lower. More than half of the patients (N = 78, 51.6%) had been diagnosed with schizophrenia for six or more years; 71.4% (N = 108) of the patients either lived with their families (meant for patients in the OPD) or were visited by them daily (meant for patients admitted in the wards). Further details are presented in Table 1.

**Table 1:** Socio-demographic characteristics of the sample Data have been represented as N and %. N = Total number of participants in the group of concern; % = percentage of participants from the total

Variable		Frequency N		Total N (%)
		Female	Male	
Age groups	18-30 years	35	46	81 (53.6%)
	31-50 years	25	37	62 (41.0%)
	51-70 years	2	6	8 (5.3%)
Ward or OPD	Ward	33	45	78 (51.6%)
	OPD	29	44	73 (48.3%)
Marital status	Single	39	59	98 (64.9%)
	Married	14	19	33 (21.8%)
	Separated	2	3	5 (3.3%)
	Divorced	2	8	10 (6.6%)
	Widowed	5	0	5 (3.3%)
Level of education	No formal education	16	18	34 (22.5%)
	Primary education	21	37	58 (38.4%)
	Secondary education	18	26	44 (29.1%)
	Higher education	7	8	15 (9.9%)
Time since diagnosis	Less than 1 year	9	6	15 (9.9%)
	1-5 years	21	37	58 (38.4%)
	6 – 10 years	16	14	30 (19.8%)
	More than 10 years	16	32	48 (31.8%)
Frequency of visits from family or friends	I live with them	25	42	67 (44.3%)
	Daily	18	23	41 (27.1%)
	Weekly	8	18	26 (17.2%)
	Monthly	3	2	5 (3.3%)
	Rarely	3	2	5 (3.3%)
	Never	5	2	7 (4.6%)
Total N (%)		62	89	151 (100.0%)

Distribution of MSPSS and PSQ-18 scores

The MSPSS scores were calculated as total scores as well as independent mean scores for family, friends, and significant others. The PSQ-18 scores were divided according to average scores from the different subsections of the scale, including technical quality, interpersonal manner, communication, financial aspects, time spent with a doctor, and accessibility and convenience. The average total MSPSS score for the sample was 39.28 (± 17.74), which reflects medium perceived support (12-35 = low, 36-60 = medium, 61-84 = high); this average is reflected by the fact that 89.4% (N = 135) of the sample had total MSPSS scores reflecting either low or moderate perceived support (MSPSS score ≤ 60). The MSPSS average scores were highest for family members (3.88 ± 1.87), indicating moderate support, whereas they were lowest for friends (2.11 ± 1.68), indicating low support. The average PSQ-18 scores were highest for interpersonal manner (3.70 ± 1.21) and lowest for general satisfaction (3.17 ± 1.09). The major differences in minimum and maximum scores among the sample demonstrate the wide level of variability among the patients (Tables 2, 3).

**Table 2:** Distribution of MSPSS and PSQ-18 scores among the sample Data have been represented as Mean±SD. Mean = the arithmetic average; SD = standard deviation (a measure of data dispersion around the mean); Maximum = the highest reported score; Minimum = the lowest reported score; MSPSS: Multidimensional Scale of Perceived Social Support; PSQ-18: Patient Satisfaction Questionnaire Short Form

MSPSS and PSQ-18 scores	Minimum	Maximum	Mean (± SD)
MSPSS average for significant other	1.00	7.00	3.81 (± 1.88)
MSPSS average for family	1.00	7.00	3.88 (± 1.87)
MSPSS average for friends	1.00	6.75	2.11 (± 1.68)
MSPSS average for total score	12.00	77.00	39.28 (± 17.74)
General satisfaction (PSQ-18)	1.00	5.00	3.17 (± 1.09)
Technical quality (PSQ-18)	1.00	5.00	3.46 (± 1.01)
Interpersonal manner (PSQ-18)	1.00	5.00	3.70 (± 1.21)
Communication (PSQ-18)	1.00	5.00	3.42 (± 1.10)
Financial aspects (PSQ-18)	1.50	5.00	3.69 (± 1.05)
Time spent with doctor (PSQ-18)	1.00	5.00	3.31 (± 1.01)
Accessibility and convenience (PSQ-18)	1.75	5.00	3.57 (± 0.82)

**Table 3:** Levels of perceived support (according to MSPSS) among the sample Data have been represented as N and %. MSPSS: Multidimensional Scale of Perceived Social Support

Level of perceived support (according to the total MSPSS score)	Frequency N (%)
Low perceived support (total MSPSS score 12-35)	61 (40.4%)
Medium perceived support (total MSPSS score 36-60)	74 (49.0%)
High perceived support (total MSPSS score 61-84)	16 (10.6%)

Association between department and gender with MSPSS and PSQ-18 scores

In order to find the association between department (ward/OPD) and gender (male/female) with MSPSS and PSQ-18 scores, a Mann-Whitney U test was conducted, which showed significant associations (indicated by p value ≤ 0.05) with department and total MSPSS scores as well as scores for family and significant other. For PSQ-18 scores, significant associations were found between the department and general satisfaction, technical quality, interpersonal manner, communication, and time spent with a doctor. Patients attending the OPD showed higher median levels of support and satisfaction compared to those in the ward for the aforementioned significant associations. As for gender, no significant associations were found for gender and MSPSS scores; however, there were significant associations between gender and PSQ-18 scores for general satisfaction, technical quality, interpersonal manner, communication, and financial aspects, with males showing higher median satisfaction levels in all statistically significant dimensions (Table 4).

**Table 4:** Association of department and gender with MSPSS and PSQ-18 scores Data have been represented as U, Z, Median Rank, p; U = Mann-Whitney U statistic; Z = standardized test statistic; Median rank = the median position of ranks assigned to each group; p-value = significance level (p ≤ 0.05* considered significant); MSPSS: Multidimensional Scale of Perceived Social Support; PSQ-18: Patient Satisfaction Questionnaire Short Form

Test/Variable		MSPSS for significant other	MSPSS for family	MSPSS for friends	MSPSS total	General satisfaction (PSQ-18)	Technical quality (PSQ-18)	Interpersonal manner (PSQ-18)	Communication (PSQ-18)	Financial Aspects (PSQ-18)	Time spent with doctor (PSQ-18)	Accessibility and convenience (PSQ-18)
N	Ward	78	78	78	78	78	78	78	78	78	78	78
	OPD	73	73	73	73	73	73	73	73	73	73	73
Median rank	Ward	63.71	66.11	73.04	66.50	58.98	65.26	64.26	62.64	72.71	61.43	73.09
	OPD	89.14	86.57	79.16	86.15	94.18	87.47	88.54	90.27	79.52	91.57	79.11
U		1888.0	2075.5	2616.5	2106.0	1519.5	2009.5	1931.5	1805.0	2590.0	1710.5	2620.0
Z		-3.58	-2.87	-0.95	-2.76	-5.01	-3.13	-3.47	-3.93	-0.99	-4.28	-0.85
p-value		0.001*	0.04*	0.340	0.006*	0.001*	0.002*	0.001*	0.001*	0.319	0.001*	0.395
N	Female	62	62	62	62	62	62	62	62	62	62	62
	Male	89	89	89	89	89	89	89	89	89	89	89
Median rank	Female	73.87	70.73	74.07	72.82	65.35	62.15	67.74	61.09	65.31	70.28	73.67
	Male	77.48	79.67	77.34	78.21	83.42	85.65	81.75	86.39	83.44	79.98	77.62
U		2627.0	2432.5	2639.5	2562.0	2099.0	1900.5	2247.0	1834.5	2096.5	2404.5	2614.5
Z		-0.50	-1.23	-0.50	-0.74	-2.53	-3.26	-1.97	-3.54	-2.61	-1.35	-0.55
p-value		0.616	0.216	0.615	0.456	0.011*	0.001*	0.049*	0.001*	0.009*	0.174	0.583

Association of other socio-demographic factors with MSPSS and PSQ-18 scores

Significant associations (indicated by p value ≤ 0.05) were found between other socio-demographic factors and MSPSS/PSQ-18 scores. Most notably, frequency of visits from family and friends was significantly associated with every subsection of both MSPSS and PSQ-18 scores. The marital status of patients was consistently associated with different subsections of MSPSS scores. Married individuals showed greater levels of support, whereas patients who reported daily visits from family and friends or those who had been living with their families displayed greater levels of both perceived support and satisfaction. Associations between age groups and overall MSPSS scores as well as MSPSS for family were statistically significant, where patients of the oldest age groups (age range: 51-70 years) reported the greatest levels of overall as well as family support. Significant relations were found between the level of education and total MSPSS as well as MSPSS for family, with patients having primary education levels displaying the highest median scores. Time since diagnosis was significantly associated with MSPSS for friends, and patients who were diagnosed in the past one to five years had the greatest MSPSS median scores. The age group, which was significantly associated with interpersonal manner, showed the greatest median scores among patients aged between 31 and 50 years old. Widowed individuals had the highest median scores for the significant association between financial aspects and marital status, but it is important to note that the extremely small sample of patients who were widowed (only five total) likely influenced these findings. Level of education as well as time since diagnosis were significantly associated with accessibility and convenience; patients of maximal education at the primary level and those who had been diagnosed for less than one year showed the highest levels of satisfaction (Table 5). 

**Table 5:** Association of other socio-demographic factors with MSPSS and PSQ-18 scores Data have been represented as H (χ²), df, p-value. H (χ²) = Kruskal-Wallis test statistic; df = degrees of freedom; p-value = level of statistical significance (p = ≤ 0.05* considered significant); MSPSS: Multidimensional Scale of Perceived Social Support; PSQ-18: Patient Satisfaction Questionnaire Short Form

Test/Variable	Groups compared	H (χ²)	df	p - value
MSPSS for significant other	Age groups	4.05	2	0.131
	Marital status	14.83	4	0.005*
	Level of education	7.08	3	0.069
	Time since diagnosis	2.58	3	0.460
	Frequency of visits from family or friends	38.84	5	0.001*
MSPSS for family	Age groups	8.04	2	0.018*
	Marital status	17.66	4	0.001*
	Level of education	10.15	3	0.017*
	Time since diagnosis	2.59	3	0.458
	Frequency of visits from family or friends	25.85	5	0.001*
MSPSS for friends	Age groups	2.52	2	0.284
	Marital status	19.69	4	0.001*
	Level of education	5.64	3	0.130
	Time since diagnosis	8.91	3	0.030*
	Frequency of visits from family or friends	17.64	5	0.003*
MSPSS total	Age groups	7.32	2	0.026*
	Marital status	18.22	4	0.001*
	Level of education	10.47	3	0.015*
	Time since diagnosis	4.15	3	0.246
	Frequency of visits from family or friends	31.51	5	0.001*
General satisfaction (PSQ-18)	Age groups	0.37	2	0.982
	Marital status	4.48	4	0.344
	Level of education	6.26	3	0.099
	Time since diagnosis	0.52	3	0.915
	Frequency of visits from family or friends	30.75	5	0.001*
Technical quality (PSQ-18)	Age groups	1.75	2	0.416
	Marital Status	4.99	4	0.288
	Level of education	3.86	3	0.276
	Time since diagnosis	2.79	3	0.424
	Frequency of visits from family or friends	25.77	5	0.001*
Interpersonal manner (PSQ-18)	Age groups	7.81	2	0.020*
	Marital status	9.36	4	0.053
	Level of education	6.01	3	0.111
	Time since diagnosis	3.32	3	0.344
	Frequency of visits from family or friends	25.49	5	0.001*
Communication (PSQ-18)	Age groups	2.03	2	0.362
	Marital status	5.23	4	0.264
	Level of education	3.84	3	0.279
	Time since diagnosis	1.28	3	0.742
	Frequency of visits from family or friends	31.90	5	0.001*
Financial aspects (PSQ-18)	Age groups	0.59	2	0.743
	Marital status	9.90	4	0.042*
	Level of education	4.49	3	0.213
	Time since diagnosis	6.71	3	0.082
	Frequency of visits from family or friends	12.98	5	0.024*
Time spent with doctor (PSQ-18)	Age groups	0.89	2	0.639
	Marital status	3.94	4	0.413
	Level of education	5.25	3	0.154
	Time since diagnosis	1.89	3	0.595
	Frequency of visits from family or friends	38.19	5	0.001*
Accessibility and convenience (PSQ-18)	Age groups	0.89	2	0.638
	Marital status	13.88	4	0.008*
	Level of education	12.63	3	0.005*
	Time since diagnosis	8.50	3	0.037*
	Frequency of visits from family or friends	16.96	5	0.005*

Correlation between MSPSS and PSQ-18 scores

In order to find the correlation between social support and patient satisfaction (i.e., MSPSS scores and PSQ-18 scores), Spearman’s rho test was used between the total MSPSS score as well as its various subsection average scores and PSQ-18 scores. Total MSPSS scores as well as average scores for family and significant others showed statistically significant positive correlations (p ≤ 0.05) with all subsections of patient satisfaction, ranging anywhere from moderate to strong correlations (ρ = 0.334 - 0.591). Average MSPSS scores for friends only showed significant correlation with interpersonal manner, which was weak to low-moderate and positive (ρ = 0.272)(Table 6).

**Table 6:** Correlation between MSPSS and PSQ-18 scores Data has been represented as ρ and p-value. ρ = Spearman’s rank-order correlation coefficient; p-value = statistical significance (p ≤ 0.05* considered significant); MSPSS: Multidimensional Scale of Perceived Social Support; PSQ-18: Patient Satisfaction Questionnaire Short Form

MSPSS measure	PSQ-18 subsection	𝜌 (Spearman’s rho)	p-value
MSPSS total	General satisfaction	0.437	0.001*
	Technical quality	0.557	0.001*
	Interpersonal manner	0.528	0.001*
	Communication	0.447	0.001*
	Financial aspects	0.374	0.001*
	Time spent with doctors	0.391	0.001*
	Accessibility and convenience	0.334	0.001*
MSPSS for significant other	General satisfaction	0.546	0.001*
	Technical quality	0.573	0.001*
	Interpersonal manner	0.459	0.001*
	Communication	0.490	0.001*
	Financial aspects	0.413	0.001*
	Time spent with doctors	0.391	0.001*
	Accessibility and convenience	0.429	0.001*
MSPSS for family	General satisfaction	0.449	0.001*
	Technical quality	0.591	0.001*
	Interpersonal manner	0.563	0.001*
	Communication	0.472	0.001*
	Financial aspects	0.378	0.001*
	Time spent with doctors	0.385	0.001*
	Accessibility and convenience	0.345	0.001*
MSPSS for friends	General satisfaction	0.029	0.723
	Technical quality	0.155	0.057
	Interpersonal manner	0.272	0.001*
	Communication	0.100	0.222
	Financial aspects	0.128	0.118
	Time spent with doctors	0.158	0.053
	Accessibility and convenience	-0.96	0.242

## Discussion

The findings of this study highlight the crucial significance of perceived social support on patient satisfaction and various associated factors between the two variables.

The noticeably lower levels of support from friends (MSPSS average for friends = 2.11) among patients demonstrate that patients suffering from schizophrenia are most likely devoid of proper friend groups or at least friend groups that they would trust. Although most other MSPSS scores remained in the moderate range, it is important to acknowledge the relatively low (N = 16, 10.6%) number of patients that demonstrated high social support levels (total MSPSS > 60). Alongside this, the fact that 40.4% (N = 61) of patients reported low social support (total MSPSS ≤ 35) signifies the need to improve support systems overall among these patients. With that being said, the fact that most patient satisfaction scores ranged in the low to high moderate range at least suggests that patients can still have a decent level of satisfaction despite a large proportion of patients having low support, possibly due to quality of care, better outcomes, and institutional support. Although it is likely that this satisfaction level could be further increased by improving the support patients receive [20].

Moreover, the significant associations with total MSPSS scores as well as MSPSS for family and significant others with department (ward/OPD) could possibly suggest that patients who are not admitted and hence are likely suffering from milder disease are more likely to have greater perceived support. Similarly, this may also reflect on satisfaction levels, with patients showing better prognosis likely being more satisfied with the treatment being provided to them [21]. It is also important to consider limited opportunities to meet family and friends in hospitalized patients. The lack of association between gender and perceived support indicates the irrelevance of gender on support levels; however, it’s interesting to see how gender differences still have an influence on various aspects of patient satisfaction, perhaps indicating better responsiveness to healthcare services based on gender, which could be due to differences in communication styles, healthcare expectations, or cultural norms [22].

The consistently significant association between visits from family and friends with MSPSS and PSQ-18 scores shows just how important social participation is when it comes to perceived support and satisfaction levels among schizophrenic patients. Although it is no surprise that marital status is significantly associated with MSPSS scores for significant others, it is rather interesting to see that MSPSS scores for friends also have a significant relation with marital status, possibly attributed to the extended social circle of patients depending on whether they are married or not. Age groups and levels of education also have a significant association with MSPSS scores for family, which shows that perceived support from the family may vary based on the age of the patient and how educated he/she is. This could be related to differences in independence among age groups and influences from educational environments. The association of time since diagnosis with MSPSS scores for friends could point towards how support from friend groups could vary depending on how long patients have been suffering from their condition, possibly due to decreased faith in patient recovery among peers [23]. The relation of interpersonal manner with age groups could signify how the emotional and personal connection that certain patients are able to build with their provider and vice versa is possibly dependent on how aged and wise the patient is through life experiences. Financial aspects and marital status could possibly be indicative of increased financial responsibilities of married individuals or even decreased financial burdens on unmarried individuals. This can be looked at both ways; perhaps it is possible that financial burdens decrease satisfaction levels due to pre-existing stress, or married individuals with better-controlled finances tend to have reduced stress. Likewise, it is possible that unmarried individuals are not experienced enough in managing finances, or alternatively, they have a reduced sense of burden. The significant relation of education level, marital status, and time since diagnosis with accessibility and convenience levels could possibly point towards an increased communication ability depending on the standard of education, improved communication with the support of a partner, and increased familiarity from experience in the event of a prolonged diagnosis, respectively [24, 25].

Moderate to strong correlation between total MSPSS scores, support from family, and support from significant others with patient satisfaction scores emphasizes the importance of perceived social support from loved ones in overall satisfaction patients experience during their treatment. This could be due to a number of things, such as assistance, emotional encouragement, reduced stress, etc. [26]. However, the lack of correlations between support from friends and satisfaction scores further points towards a lack of trustworthy friend groups among schizophrenic patients. That being said, significant correlations between perceived support from friends and interpersonal communication display that even with an overall decreased support experienced by schizophrenic patients from their friends, some degree of support may still help in enhancing patients ability to express their thoughts, concerns, and needs more effectively in social and medical interactions [27].

It is important to acknowledge the limitations of this study as well; due to the cross-sectional design of this study, we can only hope to provide a picture of social support and its correlation with perceived satisfaction at a given time, rather than finding any sort of causality between the two factors. It is also important to understand that the data is based on self-reported answers from the patients themselves, which could be subject to a couple of biases, including recall bias or response bias. Also, as this study was conducted within a specific single institution located in a low-middle income country (i.e., Pakistan) where levels of support and satisfaction may differ from other institutions throughout the world based on a number of circumstances, it is important to realize that this might affect the generalizability of the study.

This research provides significant information on the correlation between patient satisfaction and perceived social support among those with schizophrenia, utilizing evidence-based instruments within a condition-specific setting. The cross-sectional nature restricts causality interpretation but allows future studies to implement longitudinal methods for understanding temporal change. Broadening the sample across multiple institutions and geographies would enhance generalizability, and combining clinician or caregiver reports with self-reports would aid in diminishing bias. These enhancements would enhance the evidence base and enable more focused interventions in a variety of settings.

## Conclusions

This study can be used as a reference for further studies on the topic, providing insights into how social support influences patient satisfaction in patients with schizophrenia, thus guiding the development of targeted interventions to improve patient outcomes. The study highlights the role of perceived social support in influencing patient satisfaction among individuals with schizophrenia. The findings indicate that while support from family and significant others correlates with higher satisfaction levels, support from friends appears to be limited, which seems to influence its correlation with overall satisfaction. However, support from friends still has an association with satisfaction in interpersonal communication, which could suggest that enhancing social networks among friends could further improve both perceived support and satisfaction. Strengthening overall support systems remains crucial in optimizing care for schizophrenic patients.

Despite a considerable proportion of patients experiencing low social support, their satisfaction levels remained in the moderate range, possibly due to better response to treatment and quality of care. This indicates that patient satisfaction is not solely dependent on social support but can still be enhanced by strengthening support systems. Additionally, the associations between perceived support, patient demographics, and department suggest that those with milder conditions or better prognoses may have been receiving greater support and, in turn, exhibit higher satisfaction levels; alternatively, it is also possible that greater support may experience better hospital outcomes. Significant correlations between visits from family and friends with both perceived support and satisfaction scores emphasize the importance of social participation in improving patient experiences. The complexity of social dynamics in schizophrenic care is emphasized by other influencing factors as well, including marital status, education, time since diagnosis, etc. These factors also influence patient satisfaction in schizophrenics, likely due to a number of parameters, including better understanding of the condition, better communicational support, and enhanced experience.

Moving forward, interventions such as family psychoeducation, community-based programs, or implemental policies aimed at strengthening social networks and fostering supportive relationships could further improve both perceived support and patient satisfaction in schizophrenia treatment. Such interventions should be further investigated to see which function optimally to improve the relevant aspects of care among schizophrenics.
